# Durable Nanocellulose-Stabilized Emulsions of Dithizone/Chloroform in Water for Hg^2+^ Detection: A Novel Approach for a Classical Problem

**DOI:** 10.1021/acsami.2c22713

**Published:** 2023-02-23

**Authors:** Roberto J. Aguado, André Mazega, Núria Fiol, Quim Tarrés, Pere Mutjé, Marc Delgado-Aguilar

**Affiliations:** † LEPAMAP-PRODIS Research Group, University of Girona, C/ Maria Aurèlia Capmany, 61, 17003 Girona, Spain; ‡ Department of Chemical and Agricultural Engineering and Agrifood Technology, University of Girona, C/ Maria Aurèlia Capmany, 61, 17003 Girona, Spain

**Keywords:** colorimetric detection, dithizone, heavy metals, nanocellulose, Pickering emulsions, storage stability

## Abstract

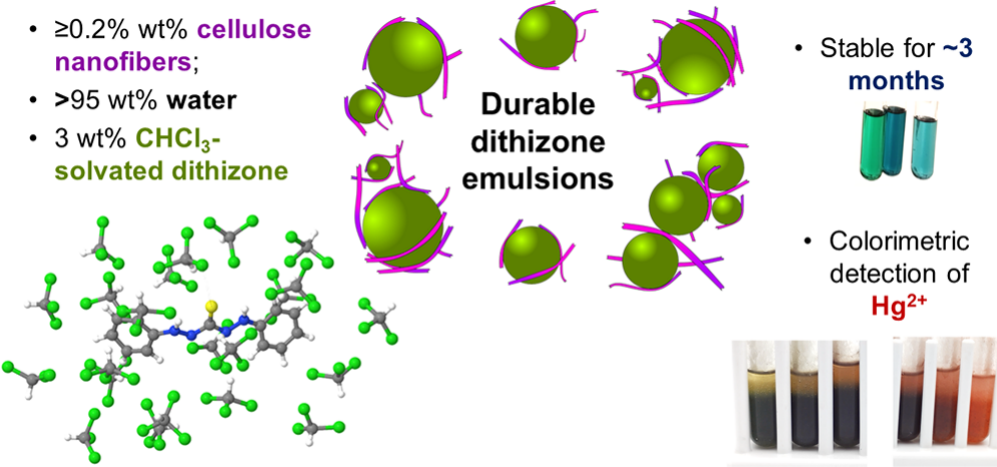

The use of dithizone (DTZ) for colorimetric heavy-metal detection is approximately one century old. However, its pending stability issues and the need for simple indicators justify further research. Using cellulose nanofibers, we attained DTZ-containing emulsions with high stability. These emulsions had water (at least 95 wt %) and acetic acid (1–8 mL/L) conforming the continuous phase, while dispersed droplets of diameter <1 μm contained chloroform-solvated DTZ (3 wt %). The solvation cluster was computed by molecular dynamics simulations, suggesting that chloroform slightly reduces the dihedral angle between the two sides of the thiocarbazone chain. Nanocellulose concentrations over 0.2 wt % sufficed to obtain macroscopically homogeneous mixtures with no phase separation. Furthermore, the rate of degradation of DTZ in the nanocellulose-stabilized emulsion did not differ significantly from a DTZ/chloroform solution, outperforming DTZ/toluene and DTZ/acetonitrile. Not only is the emulsion readily and immediately responsive to mercury(II), but it also decreases interferences from other ions and from natural samples. Unexpectedly, neither lead(II) nor cadmium(II) triggered a visual response at trace concentrations. The limit of detection of these emulsions is 15 μM or 3 mg/L, exceeding WHO limits for mercury(II) in drinking water, but they could be effective at raising alarms.

## 1 Introduction

The severity of heavy-metal pollution in developing countries is seldom correlated to their means to monitor such pollution.^1^ For instance, among the Bottom 12 countries under the category “Heavy Metals” of Yale’s Environmental Performance Index, we find Haiti, Honduras, and Guyana, three of the few American countries that do not participate in UN’s GEMStat.^2^ Especially in the absence of strong and consolidated water quality programs at national and supranational levels, it is desirable for any citizen to have ways to evaluate water quality by themselves. For instance, electrochemical probes for heavy metals can be integrated within a user-friendly sensor.^3,4^ Commercially available sensors for water pollution are generally based on electrochemical, fluorescence, infrared, or colorimetric probes.^5^ In the latter case, the sensing part usually allows for naked-eye detection in a straightforward way, even without electronic transducers.^6,7^ This is why, colorimetric systems are held in high regard for point-of-care immunoassays,^8,9^ and a similar principle could be applied to environmental monitoring.

The dangers of pollution by heavy-metal ions, their definition, and the chromogenic substances that have been tested for their colorimetric detection are extensively reported elsewhere.^10−13^ Among the known metallochromic reagents, diphenylthiocarbazone, more often known as dithizone (DTZ), is one of the most classical, most popular, and still most problematic options. It is usually assumed that its color in a given solvent result from two tautomers in equilibrium, namely, thione and thiol.^14,15^ In a recent work, Umar^14^ has indicated that the most stable conformation for DTZ is symmetrical and near-planar, with delocalized π electrons along the thiocarbazone chain. Upon chelation of divalent metal cations, each metallic atom can accept electrons from two DTZ ligands, resulting in a highly conjugated complex of tetrahedral or planar geometry.^16^ Stable complexes can also be formed between certain monovalent cations, such as Ag^+^, and DTZ anions.^17^ In all of those cases, the color of DTZ (e.g., green in acetone) changes in a quantifiable way toward orange, red, or brown.^18,19^ The different formation constants of metal dithizonates favor, conveniently from a practical point of view, some of the most worrisome metal ions, such as lead(II) or mercury(II).^20^ Nonetheless, DTZ has yet-to-be-overcome issues of photosensitivity, storability, and low aqueous solubility.

Unfortunately, the problems of DTZ in water exceed that of low solubility at pH 7 (5 × 10^–4^ g/L or lower). Once that limitation is overcome, other limitations related to instability arise.^21^ Increasing the pH enhances its solubility, but it also accelerates its rate of degradation.^22^ Likewise, surfactants or organic co-solvents do not suffice to grant chemical stability during storage, forcing measurements to be performed on the same day.^23^ While the effect of different solvent systems on tautomerism has been studied in great detail,^14,24^ a practical inquiry on their effect on storability is, to the best of our knowledge, still pending.

Two decades ago, Thiagarajan and Subbaiyan^25^ proposed an emulsion of chloroform and concentrated acetic acid, which lasts at least 3 weeks without deterioration. Our proposal involves nanocellulose to stabilize a resembling system, although with less CHCl_3_ and much less acid. Negatively charged cellulose nanofibers (CNFs) can simultaneously fulfill the roles of rheology modifier^26^ and Pickering stabilizer,^27^ which are generally closely related.^28,29^

Overall, this is the first work reporting the stabilization of DTZ/chloroform solutions in water by means of nanocellulose. We highlight the effects of the concentration of stabilizer, the physical stability of the emulsion and, not less importantly, its chemical stability in comparison to other solvent systems. Furthermore, the present work shows that emulsions are readily responsive to mercury(II) and assesses potential interferences. Finally, the plausible stabilization mechanisms and the limitations of this study are discussed.

## 2 Experimental Section

### 2.1 Materials

Bleached eucalyptus kraft pulp, unrefined (15 °SR), was provided by Ence (Navia, Spain). 2,2,6,6-Tetramethylpiperidine-1-oxy radical (TEMPO), NaBr, NaOH, NaClO (15%), copper(II) ethylenediamine, and DTZ (≥98%) were purchased from Sigma-Aldrich (Schnelldorf, Germany). Glacial acetic acid was purchased from Scharlab (Sentmenat, Barcelona, Spain). All organic solvents (reagent grade) were received from Thermo Fisher Scientific (Loughborough, U.K.). Preliminary results indicated that amylene-stabilized chloroform is preferred over ethanol-stabilized chloroform.

Distilled water was used for nanocellulose production, but metal salts were dissolved in Milli-Q water. These metal salts were lead(II) nitrate, lead(II) chloride, cadmium(II) nitrate, cadmium(II) chloride, copper(II) chloride, nickel(II) chloride, chromium(III) chloride, chromium(III) nitrate, and magnesium chloride from Panreac Applichem (Castellar del Vallès, Barcelona, Spain); potassium nitrate, iron(III) chloride, and manganese(II) chloride from Scharlab; and mercury(II) nitrate 1-hydrate, mercury(II) chloride, silver nitrate, and zinc chloride from Sigma-Aldrich.

### 2.2 Production of Nanocellulose

The bleached pulp was oxidized at 1 wt % consistency, at pH 10, with 5 mmol of NaClO as the spent oxidizer, and with 0.1 g of NaBr and 0.016 g of TEMPO per gram of fiber as co-catalysts, as described in previous works.^30,31^ The carboxyl content of the oxidized pulp, once thoroughly washed with distilled water, accounted for 0.73 ± 0.01 mmol −COOH g^–1^, as estimated by Davidson’s methylene blue adsorption method.^32^ Its intrinsic viscosity, measured by the capillary viscometer procedure (TAPPI T 230 om-08),^33^ was 2.37 dL g^–1^. From the Mark–Houwink parameters for cellulose in copper(II) ethylenediamine, as reported elsewhere,^34,35^ this corresponds to a degree of polymerization of 390.

Fibrillation was carried out in a high-pressure homogenizer, NS1001L PANDA 2 K-GEA (GEA Niro Soavi, Parma, Italy). A suspension of oxidized fibers was passed three times at 300 bar, three times at 600 bar, and three times at 900 bar. A 0.1 wt %, suspension of the resulting CNFs exhibited transmittance at 600 nm of 68%.

### 2.3 Preparation and Characterization of Pickering Emulsions

DTZ (1.7 g) was dissolved in 100 mL of chloroform at 20–25 °C. The resulting solution (3 g) was mixed with dilute aqueous suspensions of CNFs. The pH was adjusted to 5.5 by means of glacial acetic acid, requiring roughly 100–800 μL, and enough water was added to attain a total mass of 100 g. Nanocellulose consistency ranged from 0.05 to 0.60 wt %. The heterogeneous mixture was stirred with an UltraTurrax bar from IKA (Staufen, Germany), model T25, at 4000 rpm for 5 min, and left to settle within a graduated glass cylinder. The height of each phase was measured after 48 h for different CNF concentrations.

The emulsified phase had its particle size distribution measured by a dynamic light scattering (DLS) instrument, ZetaSizer Nano-ZS, from Malvern Analytical (Malvern, U.K.). For size measurements, suspended bubbles were removed by ultrasonication, KNO_3_ was added to a concentration of 10 mM, and a scattering angle of 173° (backward scattering) was set. Unlike in the case of analyzing nanofibers instead of droplets,^36^ forward scattering did not result in reproducible distributions. Furthermore, optical microscopy of stable emulsions was performed to verify that the droplet diameter of the dispersed phase in them generally was below 1 μm. The microscope was a DMR-XA model from Leica (Wetzlar, Germany), using halogen illumination and differential interference contrast.

### 2.4 Stability Studies with Dithizone in different Solvents

DTZ (7.5 μmol) was dissolved in 50 mL of each of the following solvents: ethanol, acetone, acetonitrile, chloroform, toluene, and aqueous ammonia (10%, w/v). Solutions were stored under the same conditions: at 23 °C and relative humidity 50%, in identical borosilicate glass flasks, exposed to a source of artificial light (LED, white, 36 W, 2850 lm, color temperature 4000 K), and closed with polypropylene stoppers. Flasks were only briefly open for sampling along up to 120 days. At different storage times, the electronic absorption spectrum of a sample from each solution was recorded by means of a Shimadzu spectrophotometer, model UVmini-1240. To display maximum absorbance values below 1.2, all samples, except for that in ethanol, had to be diluted. The initial dilution factor was kept constant throughout the period of exposure for each of the systems.

Among the aforementioned Pickering emulsions of DTZ/chloroform, the one whose CNF concentration was 0.20 wt % was subjected to the same stability studies, fulfilling the same storage conditions. Like in the case of ammonia, but only with the purpose of avoiding solid particles and subsequent Rayleigh scattering phenomena, DTZ was extracted and diluted with chloroform before collecting spectra at various times.

### 2.5 Computation of Solvation Shells

The recent and publicly available toolkit AutoSolvate^37^ was run in an Ubuntu 22.04 LTS environment, encompassing Python 3.7, Openbabel 2.4.0, AmberTools 22, MDTraj 1.9.4, and NGLView 3.0.3. The process involved Antechamber with the AM1-BCC model to assign point charges, LEaP for Generalized Amber Force Field parameters, and the B3LYP hybrid exchange-correlation functional for density functional theory (DFT) calculations. Both water and chloroform at 298 K were tested as solvents.

**Figure 1:**
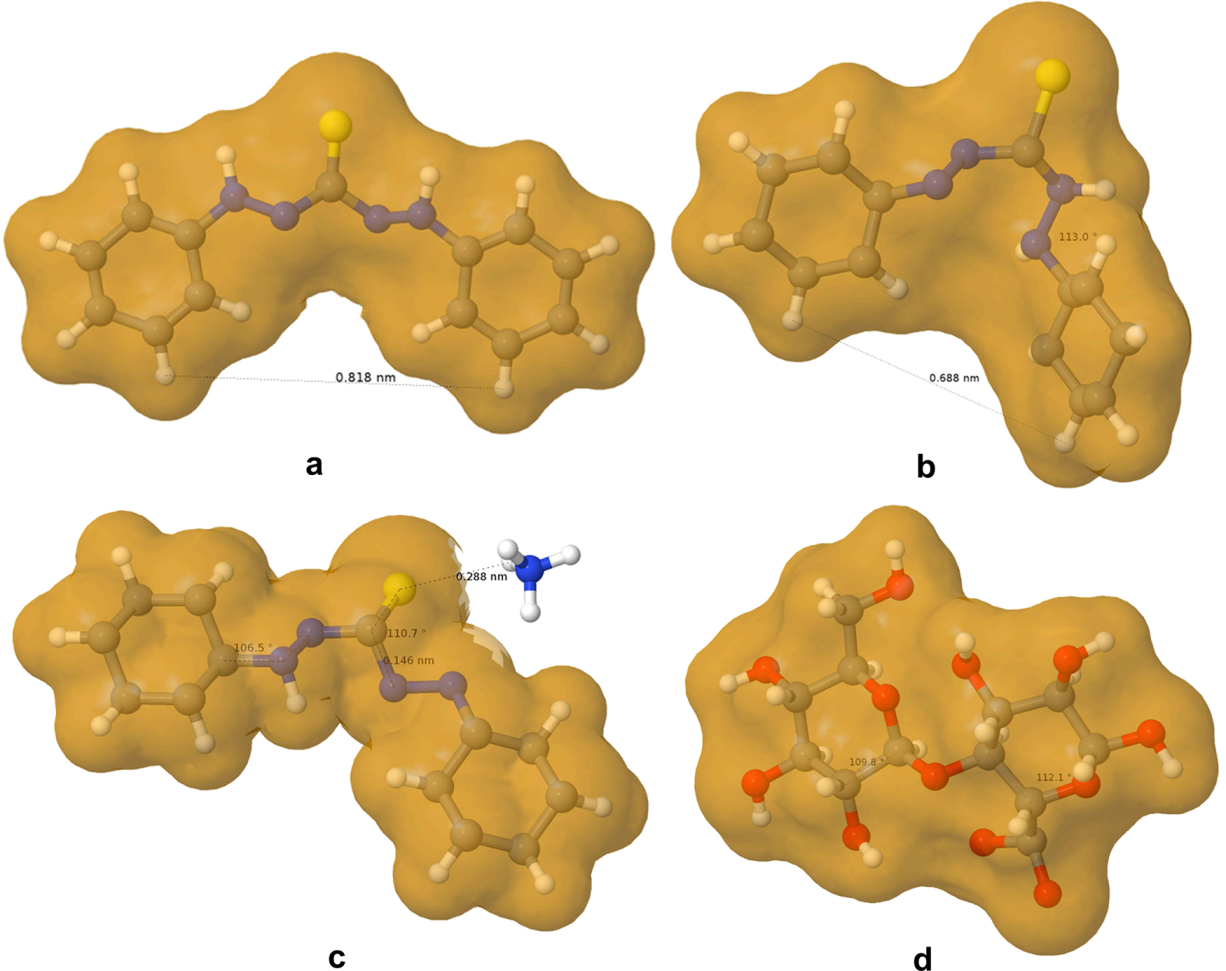
Molecular surfaces, calculated on the basis of van der Waals radii of DTZ, symmetric (a); DTZ, nonplanar thione (b); ammonium dithizonate (c); and oxidized cellobiose (d).

Three XYZ files (ESI-01) were studied for the structures of DTZ: dithizone_planar.xyz corresponds to the highly conjugated symmetric form, with the nonaromatic hydrogen atoms on the outer nitrogen atoms (Figure 1a); dithizone_thione.xyz differentiates the azo group at one side of the thiocarbazone chain and two secondary amino groups at the other side; and dithizone_anion.xyz corresponds to the deprotonated thiol tautomer of dithizone with ammonium as counterion. Finally, oxycellobiose.xyz is a proxy for CNFs, simply containing a cellobiose molecule with regioselective oxidation on carbon 6 in one of its two glucose units. Bond angles, bond distances, and molecular surfaces of these three forms were calculated and displayed by Jmol 14.

With the same toolkits (AutoSolvate, AmberTools), a simulation of molecular dynamics was run on the basis of molecular mechanical (MM) energy minimization.^38^ Then, a solvation shell considering the most neighboring solvent molecules was modeled and 10 XYZ files, also included in ESI-01, were extracted. Each file corresponds to every 10 frames, spaced by a timelapse of 4 ps, of the dynamic simulation.

### 2.6 Response to Heavy-Metal Salts

Emulsions containing 0.20 wt % CNFs, 3 wt % DTZ/chloroform, and 0.4 vol % acetic acid were mixed in a 1:9 metal solution-to-emulsion ratio (v/v) with different metal salts in aqueous solution. The blank was prepared with Milli-Q Lab Water. Additional tests involved tap water (Girona, Spain) and a sample of natural water from Ter River (41.991°N, 2.819°E, 21 November 2022). The limit of detection (LOD) was estimated as the lowest concentration tested whose colorimetric response, expressed as sRGB coordinates, differed significantly from that of the blank
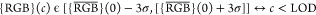
1where *c* is the concentration of the metal salt and σ is the standard deviation of the blank.

Then, Hg(NO_3_)_2_ was chosen for quantification purposes. For that, the aforementioned emulsions were combined, once again in a 1:9 ratio (v/v), with solutions of Hg(NO_3_)_2_ of increasing concentration.

### 2.7 Colorimetric Assays

Sampling was performed in 1.5 cm wide glass assay tubes. Although the color change was immediately noticeable, the high viscosity of the emulsion slowed down the diffusion of metal ions. Therefore, tubes were briefly shaken (2–3 s) on a tabletop vortex mixer, as shown in the video (ESI-02) and left to settle for 60 min. Color coordinates were extracted in two ways: by photography, using a smartphone, and by means of an X-Rite portable device, model RM200 (Grand Rapids, MI).

Photographs were taken after placing the emulsions on a test tube rack and into a LED-illuminated light box (20 W, white, color temperature 6000 K). The γ correction of the smartphone’s camera under these conditions of surface luminance (310 cd m^–2^) was approximately 2.2. To remove color dominance, the tool Color Balance was used so as to adjust the background to {180, 180, 180}. Mean sRGB color coordinates over a reflection-free rectangular area were computed with the open-source platform ImageJ, using the plugin RGB Measure. Parallelly, the X-Rite device readily reported the CIE 1976 L*a*b* coordinates of a suspension placed in a cuvette with an optical path length of 10 mm.

## 3 Results and Discussion

### 3.1 Solvatochromism of Dithizone

**Figure 2:**
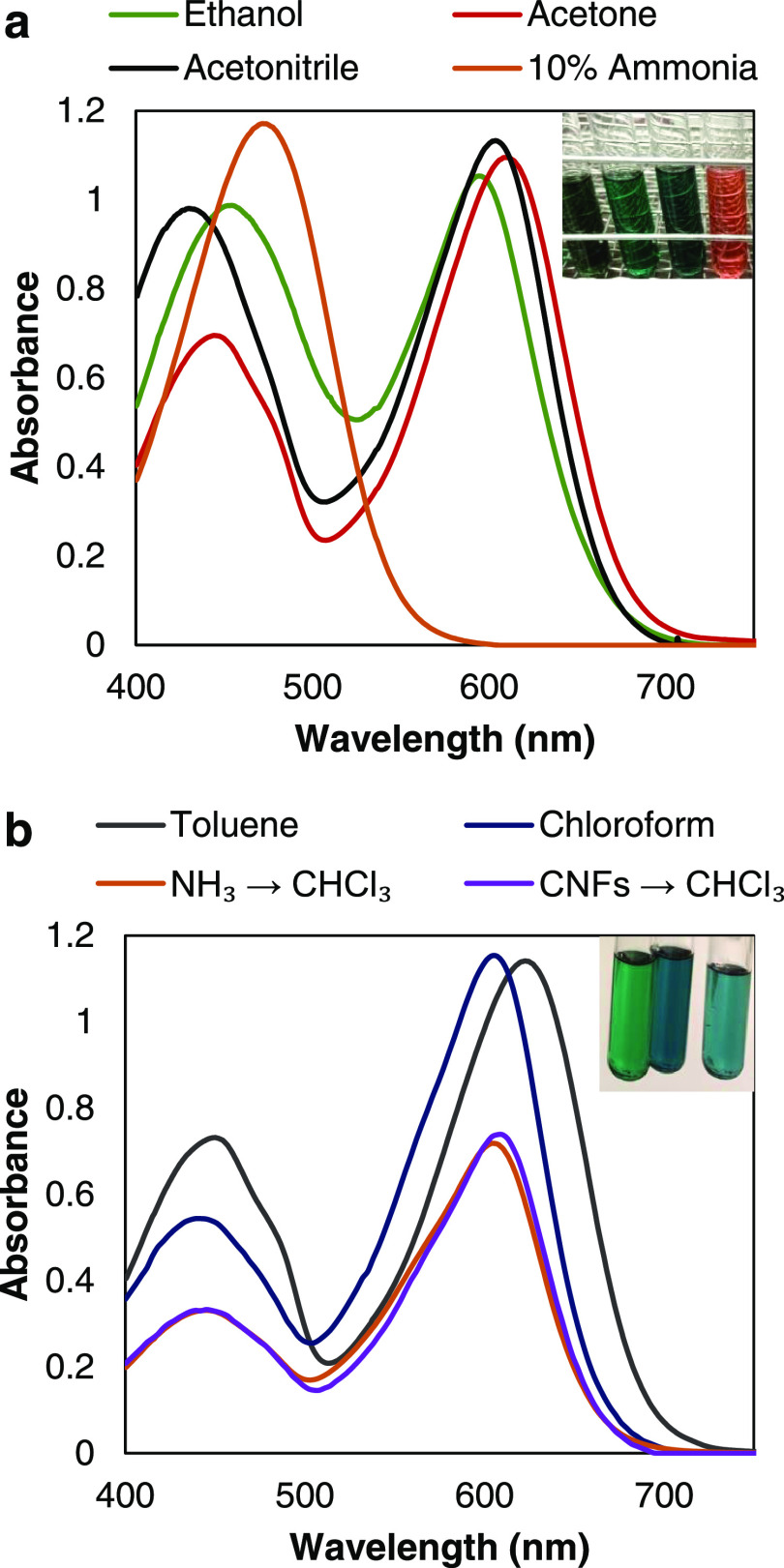
Electronic absorption spectra of DTZ in different solvents, regarded as polar (a) and nonpolar (b). Inset in (a), from left to right: ethanol (0.15 mM), acetone (0.15 mM), acetonitrile (75 μM), aqueous NH_3_ (80 μM). Inset in (b), from left to right: toluene (55 μM), CHCl_3_ (40 μM), and CHCl_3_ extracts from aqueous systems.

Initially, DTZ solutions in organic solvents at concentrations around 0.15 mM are green, greyish green, cyan, or blueish. In all of these cases, corresponding to different tautomeric equilibria, DTZ is readily responsive to heavy-metal ions. Figure 2 displays the initial absorption spectra in the visible region, each corresponding to the average of two equally prepared solutions. Generally, solutions showed strong absorption at the band generally assigned to the thione form (595–625 nm), except in the case of ammonia (Figure 2a). As known, DTZ is practically insoluble in acidic aqueous media,^39^ but soluble in alkalis. Nonetheless, the equilibrium in this case is shifted to the dithizone anion (Figure 1c), making the solution appear orange. This is reversible, at least during the first hours, as a green solution is obtained by acidifying and extracting the DTZ with an immiscible solvent (Figure 2b), or even by acidifying and adding a miscible organic co-solvent. Thus, for as long as the solution was not completely degraded, a liquid–liquid extraction was performed with chloroform to check that the characteristic green/cyan color could be recovered. The absorbance for a completely degraded DTZ/ammonia solution or its acetic acid/chloroform extracts was close to 0.

The solubility of DTZ in CHCl_3_ is likely higher than in all other solvents, polar or not, reported so far: 0.04 g/L in hexane, 0.95 g/L in toluene, 0.03 g/L in ethanol, 0.9 g/L in acetone, 1 g/L in acetonitrile.^39^ With a molar absorption coefficient of 29 mM^–1^ cm^–1^ (this work), even higher than that of DTZ/toluene, another advantage lies in needing less weight of DTZ to display a certain color intensity. In another context, chloroform extracts from the nanocellulose-stabilized emulsion qualitatively exhibit similar absorption spectra to those of DTZ/CHCl_3_, but with a certain bathochromic shift arising from the presence of water. Evidently, chloroform extracts from aqueous systems, either fresh DTZ/ammonia or DTZ/CNFs, were saturated in water, amounting to 0.076 wt % of water at 20 °C.^40^

**Figure 3:**
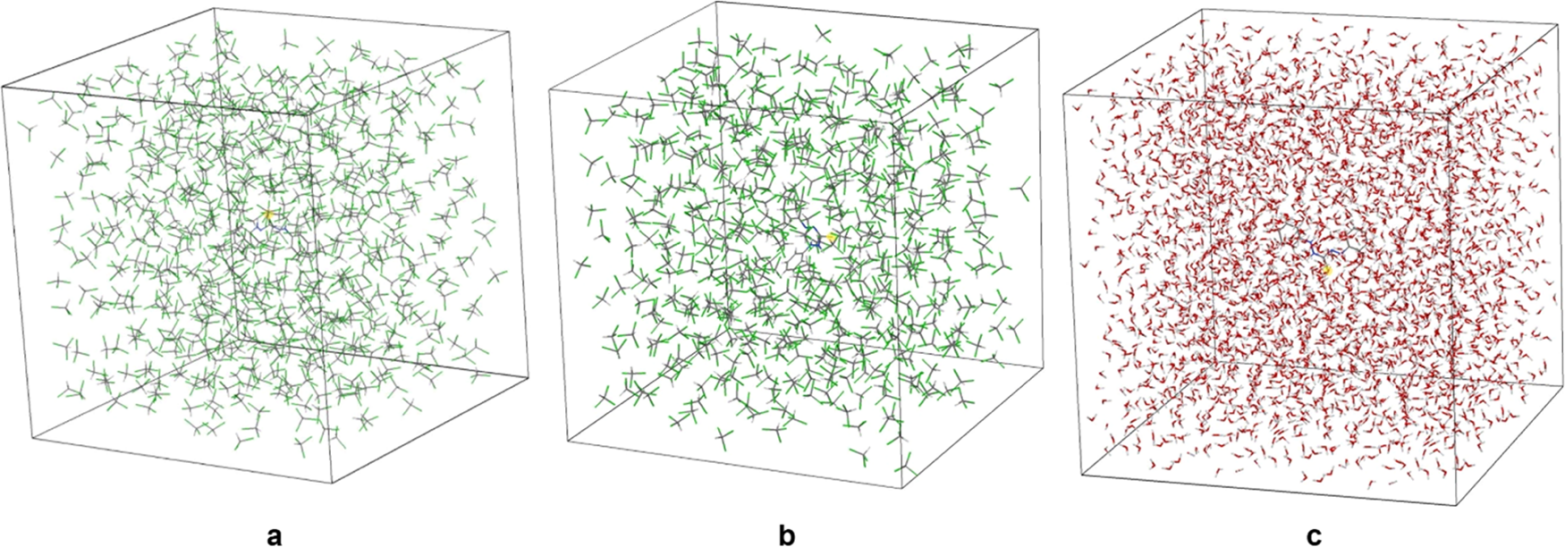
Computed solvation boxes (3 nm × 3 nm × 3 nm) of symmetric DTZ in chloroform (a); nonplanar thione form in chloroform (b), and DTZ anion in an alkaline aqueous medium (c).

The solvation box of DTZ in chloroform is significantly dependent on the conformation. The broader the dihedral angle between the two sides of the thiocarbazone chain, the more solvent molecules were estimated to be in the vicinity of the solute (Figure 3). The number of chloroform molecules in 27 nm^3^ is 636 in Figure 3a, corresponding to the most stable conformation.^14^ The same volume allocates 524 solvent molecules in the case of the asymmetric thione form. It should be noted that, at this point, the effects of the solvent on the conformation of the solute are not considered. Figure 3c displays the solvation of the DTZ anion in water, accounting for 2960 solvent molecules in 27 nm^3^.

### 3.2 Stability of Dithizone in Different Solvent Systems

**Figure 4:**
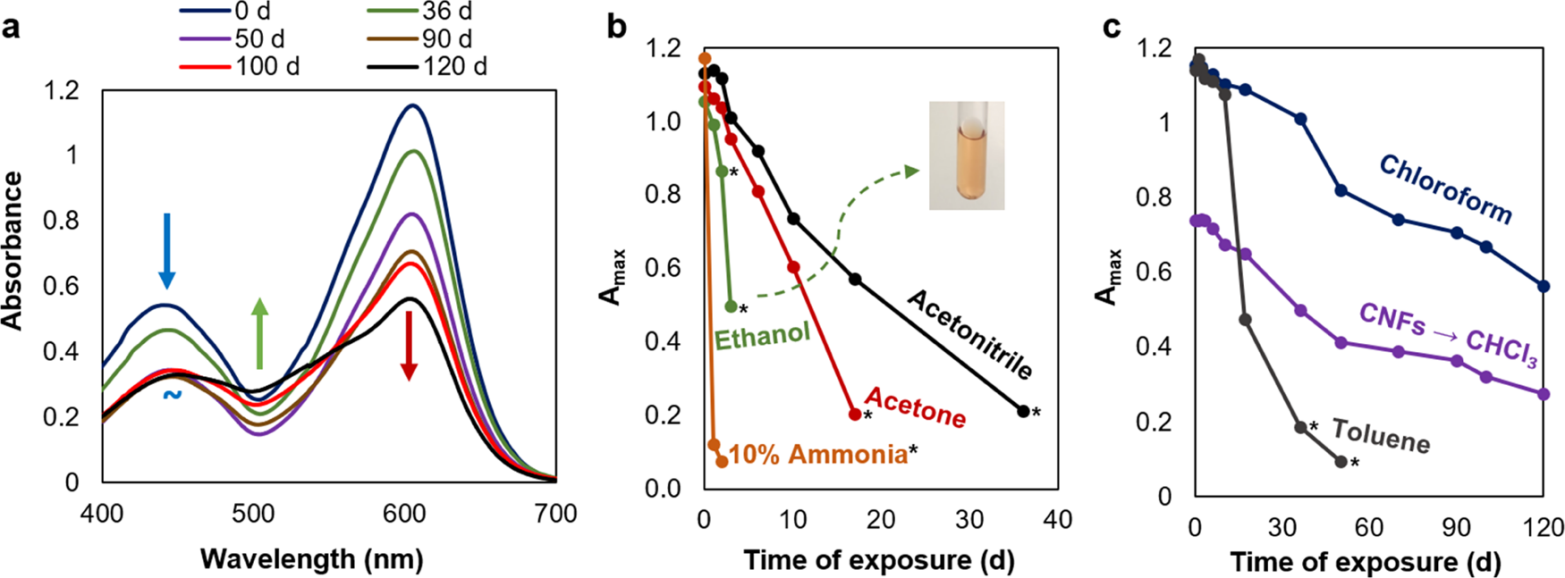
Evolution of selected absorption spectra of DTZ/chloroform (a); diminishment of the maximum absorbance in polar solvents (b) and in more stable systems (c).

DTZ was remarkably more stable in amylene-stabilized chloroform than in any other solvent, as evidenced in Figure 4. Even in the case of chloroform (Figure 4a), there was progressive reduction of the maximum molar absorptivity assigned to the thione form (604 nm) from the first days. The band associated with the thiol form (452 nm) decreased during ca. the first 50 days, but then it remained an isosbestic point. In addition, from that moment on, the absorbance in the cyan-to-green region started to increase, probably due to a degradation byproduct. While some dithizonates display photochromism, the absorption band of the resulting isomers is found at longer wavelengths, ca. 620 nm.^18,41^ Nevertheless, despite the effects of aging, the system was functional for colorimetric purposes even after four months. Likewise, the CNF-stabilized emulsion took 3 months to lose roughly half of its maximum absorbance and resonance was not harmed.

Overall, the storage stability of dithizone in these different solvent systems follows this sequence, from more to less stable: chloroform ∼ chloroform/acetic acid/CNFs > toluene ∼ acetonitrile > acetone > ethanol > aqueous ammonia. The DTZ/ammonia solution was severely degraded after 24 h, likely due to the deprotonated thiol being strongly nucleophilic and thus chemically unstable. In Figure 4b,c, an asterisk (*) indicates that the maximum absorbance corresponded either to the thiol form or to a degradation product with prominent absorption bands between 400 and 500 nm. In these cases, the color approached that of the inset image (orange), which depicts degraded DTZ/ethanol. At this point, the usability of DTZ for the colorimetric detection of Hg^2+^ detection is null or severely impaired.

Regarding the DTZ/ClCH_3_/CNFs/acetic acid/H_2_O system, it is worth noting that the addition of any organic co-solvent is counter-productive. Rauf et al.^24^ indicated that in a mixture of miscible solvents, DTZ molecules are solvated by the most polar one. In the aforementioned Pickering emulsion, DTZ is solvated by CHCl_3_ because of its insolubility in aqueous acidic media. While a co-solvent could stabilize the system without the aid of nanocellulose, it would defeat the purpose of molar absorptivity and stability.

As a side result, diluted DTZ/chloroform solutions (e.g., 40 μM) were less stable if chloroform was stabilized with ethanol. Likely, in such cases, DTZ was preferably solvated by the most polar solvent in the mixture. This could be avoided by shaking the liquid in the presence of anhydrous CaCl_2_,^42^ as long as chloroform is then kept away from ultraviolet radiation.

**Figure 5:**
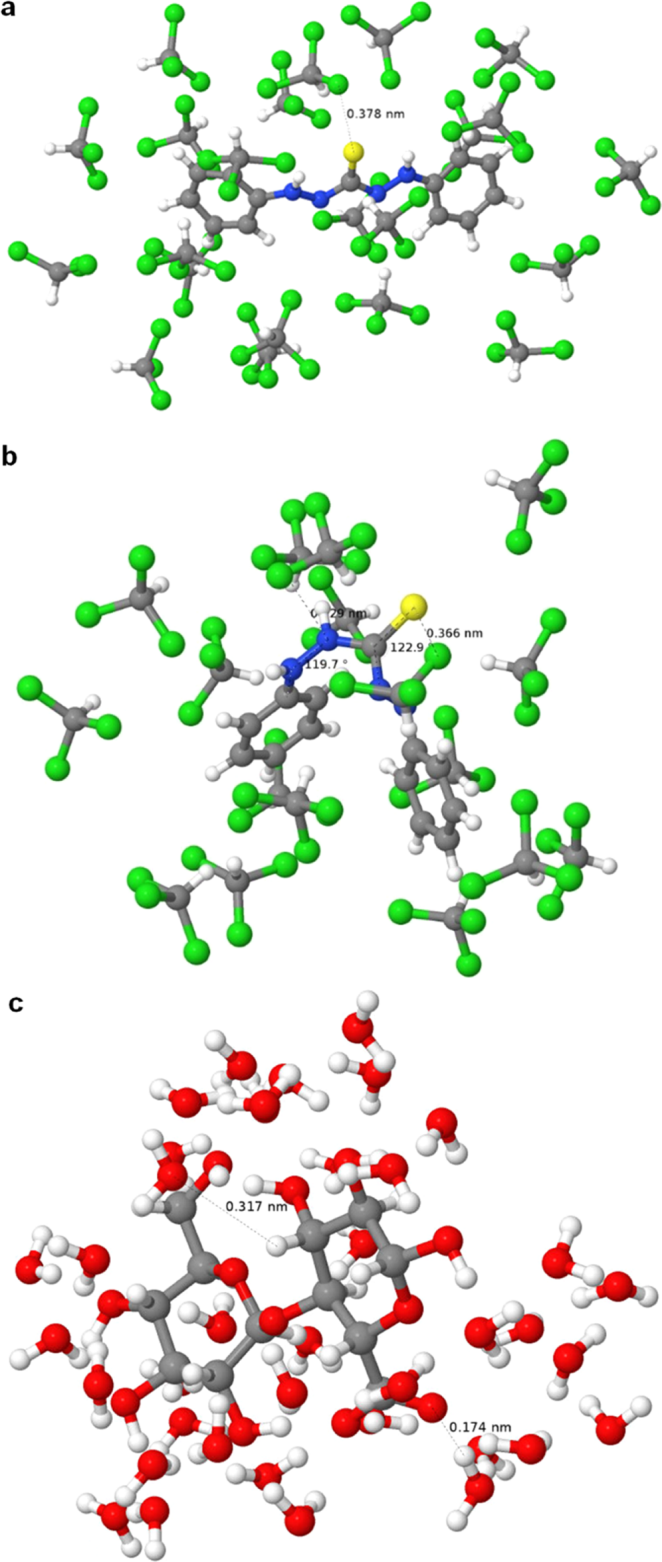
Solvation clusters of symmetric DTZ/chloroform (a), asymmetric DTZ (thione)/chloroform (b), and oxidized cellobiose/water (c).

Molecular dynamics of the solvation of DTZ in chloroform estimated that, by inducing out-of-plane rotations on the thiocarbazone chain, the angle between the phenyl rings was slightly reduced. In the frame presented in Figure 5a for symmetric DTZ, which is the most stable conformation, this angle went from 180 to 169° upon solvation. This folding was more pronounced in the case of the asymmetric thione (Figure 5b), nearly forming a right angle (∼90°). Moreover, it can be noticed that chloroform molecules orient their chlorine atoms toward the sulfur atom of DTZ. Overall, one DTZ molecule is closely surrounded, with intermolecular distances of 4 Å or less, by 22 chloroform molecules in its symmetric conformation, and by 16–18 molecules in its less stable asymmetric form. Hypothetical cluster calculations of oxidized cellobiose in chloroform (ESI-01), as proxy for CNFs, indicated that VDWAALS energy was the main contributor to stability (−14.5 kcal/mol). Not surprisingly, the solvation of CNFs by water (Figure 5c) is much more thermodynamically favorable. Interactions with water are stronger and with shorter interatomic distances along the equatorial directions (O–H···O) than on the axial planes (C–H···O).

### 3.3 Pickering Stabilization: Effect of Concentration

**Figure 6:**
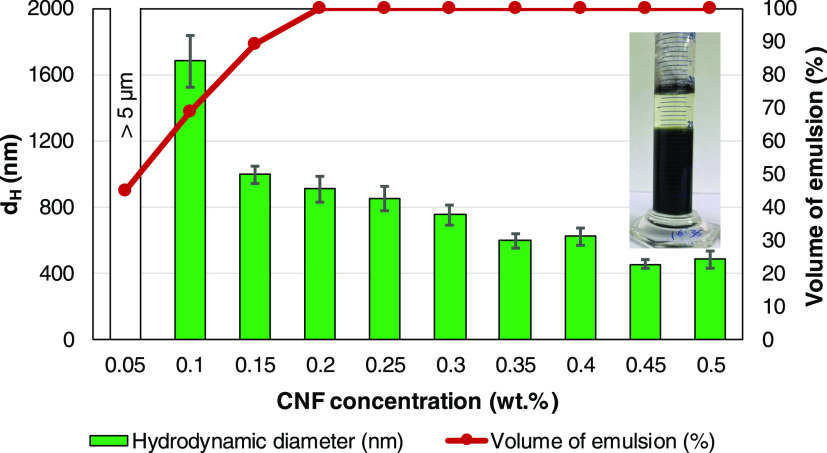
Hydrodynamic diameter, as estimated by DLS, and percentage of the volume of the cylinder occupied by the emulsified phase, as a function of the concentration of nanocellulose. The inset exemplifies the case for 0.10 wt % CNFs.

The DTZ/chloroform phase, accounting for only 3 wt % of the mixture, was easily emulsified in acidic aqueous media with CNFs. Even after weeks of storage, the mixtures contained no purely organic phase (or nonemulsified oil) at all. The other phase (serum) accounted mainly for CNFs and water. Its height decreased with the concentration of stabilizer, as commonly found in most reports on Pickering stabilization.^43,44^ For CNF consistencies equal to or higher than 0.20 wt %, no serum phase was appreciated and the whole mixture was emulsified. This is shown in Figure 6, along with the intensity-average hydrodynamic diameter (*d*_H_) of organic dispersed droplets.

It could be stated that these mixtures were not physically stable during the first 10–30 h. Nonetheless, at some point, aggregation reached an apparent equilibrium and the interphase between the emulsion and excess water became clear. *d*_H_ measurements correspond to this stage. However, it is worth mentioning that, simply with concentrations above 1% of particles whose size is greater than 100 nm, the measuring conditions do not meet the recommendations of the manufacturer. However, any dilution would change the system and, at the very least, these results are useful for comparative purposes. In this sense, it is clear that, upon increasing the consistency of the stabilizer (CNFs), droplet size decreased, although this diminishment was slight or even nonsignificant in the high end of the concentration interval.

**Figure 7:**
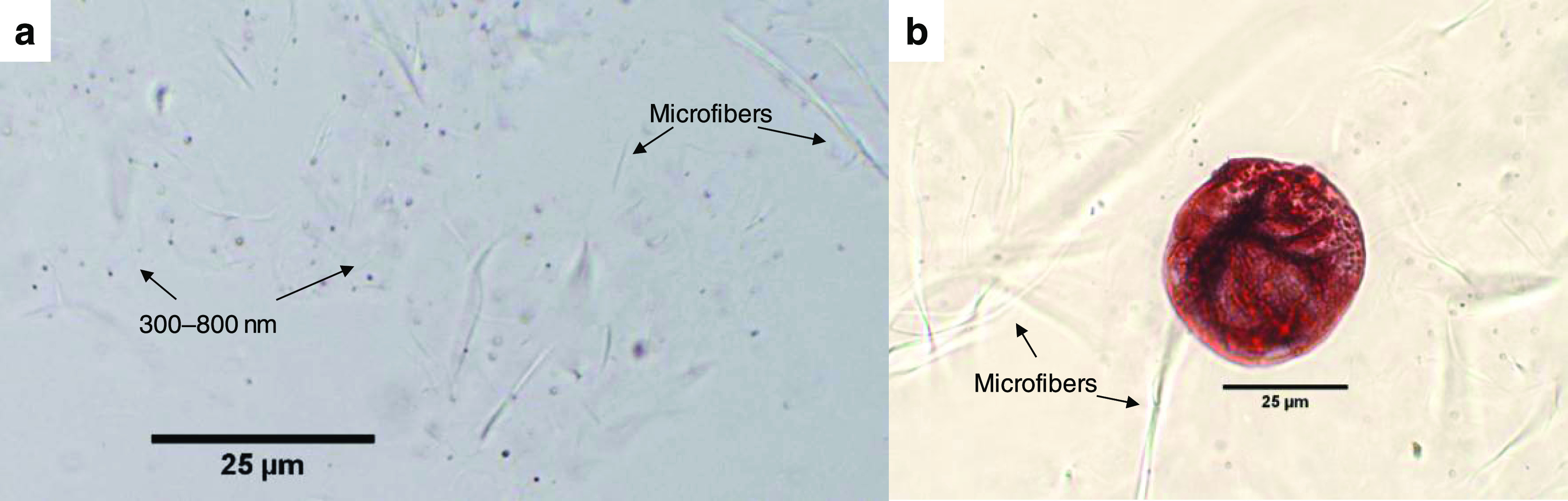
Micrographs of Pickering emulsions: DTZ/chloroform (3 wt %)/nanocellulose (0.30 wt %)/acetic acid/water (a), and the same system with Ag^+^ (b).

Although not attaining enough resolution to obtain accurate droplet size distributions, the micrographs in Figure 7 are mostly consistent with *d*_H_ estimations. Most of the measurable droplets (with ImageJ, for instance) are 0.3–0.8 μm in Feret’s diameter (Figure 7a). In another context, the aggregate particle shown in Figure 7b was obtained by adding silver nitrate up to a 1 mM concentration. Mercury(II) was not chosen for this task for safety reasons. Besides the evident color change to red, the heterogeneity of the particle seems to indicate the presence of nanofibers, air, and even reduced silver. It should be noted, in any case, that the fibrous elements distinguished in these micrographs are more precisely described as microfibers (diameter > 100 nm) that remain after high-pressure homogenization.^45,46^ Rigorously speaking, nanofibers cannot be visualized at these magnifications.

### 3.4 Colorimetric Detection of Heavy Metals by Pickering Emulsions

**Figure 8:**
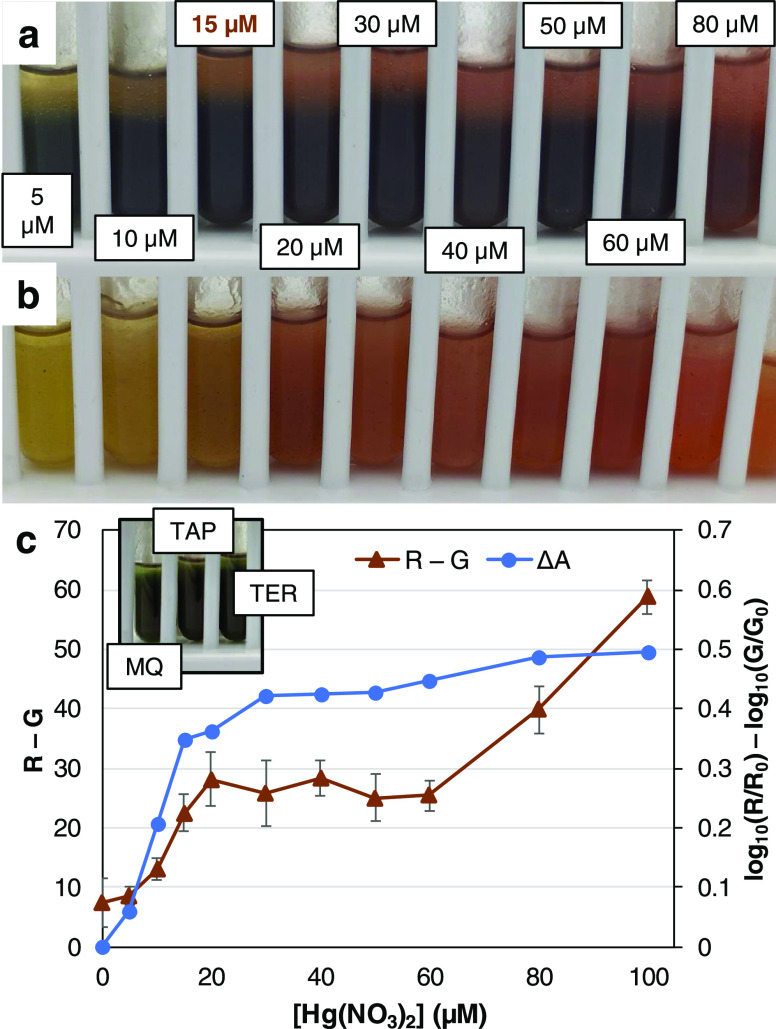
Optical response of nanocellulose-containing DTZ/chloroform emulsions to mercury nitrate: photograph of the system proposed (a), a side experiment with a lower concentration of DTZ (b), and two sRGB-based functions (c). Intervals encompass twice the standard deviation. The inset corresponds to the water samples.

Since the system proposed in this work aimed at carrying as much dithizone as possible while keeping the water content above 95 wt %, the emulsions displayed high absorbance if the optical path was long enough. This is the case of the assay tubes in Figure 8a. Alternatives for quantifying the optical response from the emulsified phase may be as simple as choosing thinner tubes or decreasing DTZ concentration (example in Figure 8b). Nonetheless, we took advantage of the cloudy aqueous phase that appeared due to the dilution of CNFs below 0.20 wt %, and thus Figure 8c plots color parameters of this phase against the concentration of Hg(NO_3_)_2_. It can be noted that, by increasing Hg(II) concentration toward the millimolar range, the emulsion turns purple (SI), due to changes in metal–DTZ coordination.^18^

In any case, it was evident that, in comparison to the blank (Figure 8c, inset), the addition of Hg(NO_3_)_2_ decreased the green coordinate and increased the red one. The simplest approach to express this as a measurable function is the R – G difference. The subsequent computation required is minimal and even common smartphones could be used for the task.^47,48^

Nonetheless, often with the intention of applying Beer’s law, some works on colorimetric detection use an analogy of sRGB coordinates to the absorbance at complementary colors.^6,49^ For example, for red
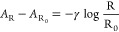
2where *A*_R_ would be the maximum absorbance at wavelengths below 550 nm (a rough approximation), the subscript 0 refers to the blank, and γ is the γ correction. Figure 8b, besides R – G, also considers *A*_R_*– A*_G_, both calculated as in eq 2.

**Figure 9:**
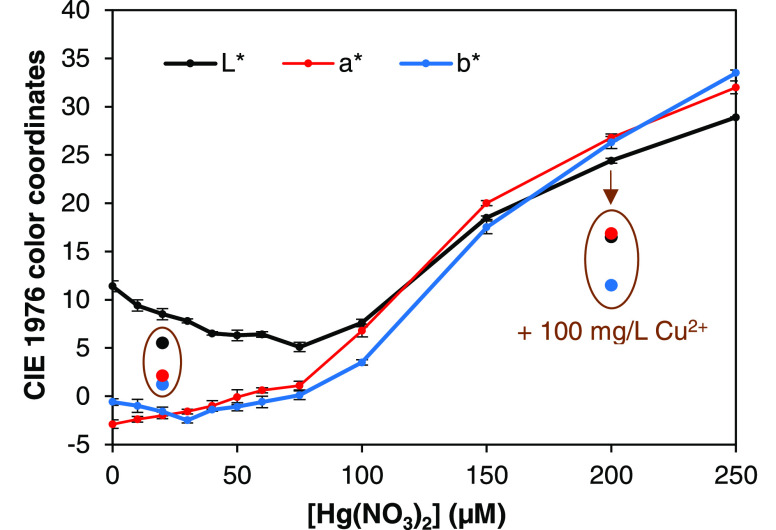
Results from the X-Rite colorimeter: coordinates in the CIE 1976 L*a*b* color space as a function of the concentration of mercury(II) nitrate. Isolated points in brown ovals show the interfering effect of 100 mg/L CuCl_2_ at different concentrations.

While the function is monotonously increasing with Hg(II) concentration and it is useful for computation, it tends to level off even if R – G increases. Furthermore, the quantification between 15 and 80 μM is not reliable. These limitations belong to the indirect detection system, based on photography and image treatment, not to DTZ itself.^50^ Indeed, direct measurements with the colorimeter, which ensures consistent conditions in terms of luminance and distance to the sample, achieved much lower random errors, as shown in Figure 9. The three coordinates (L*, a*, b*) change their trend with the concentration of Hg(NO_3_)_2_ at 75–100 μM.

Like R – G, but not equivalently, the coordinate a* also indicates to what extent the sample reddened with increasing concentration of mercury(II). In fact, it was the only coordinate found to be monotonously increasing. At least up to 75 μM, the concentration of mercury(II) nitrate could be linearly fitted (*R*^2^ = 0.980) to Δa* (i.e., a* – a*_0_)
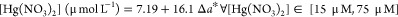
3A linear trend of [Hg(NO_3_)_2_] with the optical response has also been reported for DTZ/chloroform without CNFs.^50^Figure 9 also displays the effects of an interfering salt, CuCl_2_, on colorimetric measurements. In the range described by eq 3, the presence of 100 mg/L CuCl_2_ reddened the sample to a larger extent than estimated by said equation. In contrast, for a high concentration of Hg(NO_3_)_2_ (200 μM), 100 mg/L CuCl_2_ had a negative interfering effect. A similar phenomenon was found with NiCl_2_.

It can be stated that the system was more responsive toward Hg(II) than to any other metal tested, but not that the system was selective toward mercury(II) salts. Besides CuCl_2_ and NiCl_2_ (toward brown), at least AgNO_3_ (reddening) and ZnCl_2_ (toward pink) were identified as interfering salts at trace concentration. Common noninterfering salts and ions that could jeopardize the estimation of mercury(II) concentration by eq 3 are shown in Table 1.

**Table 1:** Values or Intervals for the Limit of Detection of the DTZ/Chloroform (3 wt %)/Nanocellulose (0.20 wt %)/Acetic Acid/Water Emulsion toward Different Metal Salts

salt	LOD (mg/L)	salt	LOD (mg/L)
KNO_3_	noninterfering	ZnCl_2_	40–50
NaCl	noninterfering	AgNO_3_	20–30
MgCl_2_	noninterfering	CdCl_2_	>100
CrCl_3_	>100	Cd(NO_3_)_2_	>100
MnCl_2_	>100	Hg(NO_3_)_2_	3
FeCl_3_	noninterfering	HgCl_2_	5–10
NiCl_2_	80–100	Pb(NO_3_)_2_	>100
CuCl_2_	60–80	PbCl_2_	>100

The fact that mercury(II) nitrate has less covalent character than the corresponding chloride (poorly soluble, poorly conductive) explains why the LOD was lower for the former (15 μM or 3 mg/L) than for the latter (5–10 mg/L, Table 1). Unexpectedly, both Cd^2+^ and Pb^2+^, whose association constants with DTZ are also high,^20^ did not alter the color of the emulsified or serum phases at concentrations below 100 mg/L. Possibly, some metal ions became associated to a significant extent to the carboxylate groups of CNFs, which prevented them from accessing the sulfur atom of DTZ. In contrast, Hg^2+^, as a paradigmatic soft Lewis acid, was unlikely to accept electrons from oxidized CNFs.

It is also worth noticing that neither the compounds and microorganisms naturally present in Ter River (Figure 8c, inset), nor those present in tap water (chloramines, carbonates, etc.), interfered with the optical response.

### 3.5 Mechanism and Limitations

In most works on emulsions with nanocellulose, stabilization is described in terms of the yield stress (rheology) and/or due to the hydrophobic effect.^31,51^ Since even at very low CNF concentrations (0.05 wt %), there was no layer of excess DTZ/chloroform, it may be argued that rheological stabilization was not the only mechanism involved.

It is consuetudinary to speak in terms of “hydrophobic interactions” between the oil phase and the cellulose chains, and among the cellulose chains in each of the fibrils. It is probably more accurate to express them as dispersive interactions that, in the presence of water, take place in areas excluded from hydrogen bonding. This includes the surfaces of cellulose that are parallel to the (200) plane since its O–H bonds are equatorial. Hence, even though cellulose as material is mostly hydrophilic and oxidized nanofibers are still more prone to hydration, they have surfaces that will not H-bond with water. According to recent advances on the molecular dynamics of cellulose aqueous media, fibrils untwist to maximize interaction with those surfaces when adhering to hydrophobic materials.^52^

Most applications of nanocellulose as Pickering stabilizer involve alkanes, vegetable oils, and several sorts of long-chain aliphatic compounds as the organic phase.^27,31,43^ However, in our case, chloroform has a dipole moment of 1.15 D and it is a fair hydrogen bond donor.^53^ Although the aforementioned simulation of a chloroform-solvated oxidized cellobiose molecule returned a positive value for free energy, dipole–dipole interactions were the highest contributors to energy minimization. Furthermore, strong water–chloroform interactions at the interface of both liquids have been reported.^54^ All considered, chloroform–CNF–water interactions are complex phenomena that should not be approached from a reductionist point of view.

However, besides this academic interest on cellulose–chloroform interactions, the applicability of durable nanocellulose-stabilized Pickering emulsions at a large scale requires chloroform to be replaced by a less hazardous solvent. Furthermore, it may be pointed out that the LOD for Hg^2+^ lies 3 orders of magnitude above the limits of both U.S. EPA and WHO guidelines for drinking water,^55^ although the proposal is still useful, for instance, to raise alarms regarding certain industrial effluents or spills. If key limitations were overcome, emulsions could be integrated within optical sensing devices. Other applications, such as film forming and paper coating toward responsive strips (ESI-02), could as well be developed.

## 4 Conclusions

We herein report noteworthy findings, some of them unexpected, on the Pickering stabilization of DTZ/chloroform solutions using nanocellulose. Despite the hazardous nature of chloroform, its solvation of dithizone (22:1 in a 4 Å radius, as calculated by energy minimization) keeps it functional during lost storage times. Furthermore, DTZ in chloroform attains high solubility (17 g/L at 20 °C) and high molar absorptivity (29 mM^–1^ cm^–1^). An emulsion of DTZ/chloroform (3 wt %) in water with 0.4 vol % acetic acid and 0.2 wt % CNFs was functional during at least three months of storage, when it had lost roughly 50% of its maximum absorbance. It did not display phase separation and it was immediately responsive to mercury(II) nitrate at concentrations of 15 μM (3 mg/L) or higher. Interestingly, the LOD for Pb^2+^ and Cd^2+^, which are also known to form colored complexes at trace concentration, was beyond 100 mg/L.

On the one hand, nanocellulose-stabilized emulsions offer an interesting answer to the old issue of attaining an effective and durable dispersion of dithizone in water. On the other hand, in the context of Hg^2+^ analysis, the colorimetric method offered by these emulsions is less sensitive than, for example, atomic absorption spectroscopy or inductively coupled plasma mass spectrometry. Optimization studies are needed to lower the LOD, improve quantification, and, if possible, replace chloroform while still attaining long-lasting stability.

## Supporting Information Available

Additional figures, photographs, and characterizations, including thermograms and FTIR spectra (PDF)
XYZ/PDB files, either inputs or outputs for MM calculations (ESI-01) (ZIP)
Illustrative video (ESI-02) (MP4)

## Abbreviations

CNFs: cellulose nanofibers
d_H_: hydrodynamic diameter
DFT: density functional theory
DLS: dynamic light scattering
DTZ: dithizone
MM: molecular mechanical
sRGB: standard red, blue, and green color space
TEMPO: 2,2,6,6-tetramethylpiperidine-1-oxyl radical
WHO: World Health Organization
