# Supplementary material for: Durable Nanocellulose-Stabilized Emulsions of Dithizone/Chloroform in Water for Hg2+ Detection: A Novel Approach for a Classical Problem
Source: ACS Appl Mater Interfaces. 2023 Feb 23;15(9):12580–9. doi: 10.1021/acsami.2c22713 (PMC9999351; doi:10.1021/acsami.2c22713)
Supplement: Supplementary file 1 — am2c22713_si_001.pdf [file am2c22713_si_001.pdf]

Supporting Information (SI)

Durable Nanocellulose-Stabilized Emulsions of Dithizone/Chloroform in Water for Hg^{2+} Detection: A Novel Approach for a Classical Problem

Roberto J. Aguado[1,2,], André Mazega[1,2], Núria Fiol[2], Quim Tarrés[1,2], Pere Mutjé[1,2], Marc Delgado-Aguilar[1,2]*

[1]LEPAMAP-PRODIS Research Group, University of Girona, C/ Maria Aurèlia Capmany, 61, 17003 Girona, Spain;

[2] Department of Chemical and Agricultural Engineering and Agrifood Technology, University of Girona, C / Maria Aurèlia Capmany, 61, 17003 Girona

* roberto.aguado@udg.edu

S1. Emulsion-metal phenomena

Figure S1 displays photographic evidence of metal-emulsion interactions that, albeit left out of the article, can be of interest. In Figure S1a, the two assay tubes at the left correspond to mercury(II) nitrate concentrations of 0.2 mM (second-to-last one) and 1 mM (last one).

Figure S1b reveals that selectivity towards mercury(II) is actually possible with a variation of the DTZ/chloroform (3%)/acetic acid/CNFs/water, since the only systems that reddened at trace concentrations were those with $HgCl_2$ and $HgNO_3$. This was achieved simply by reducing the amount of DTZ. However, it implied increasing the LOD not only for Ag^+, Zn^{2+} and the rest, but also for Hg(II) salts too.

Figure S1c shows that, by adding a metal salt (Ni^{2+} in this case), the yield stress decreases to the point that nanocellulose-stabilized emulsions no longer stand as gels. However, the emulsion was not disrupted, which supports our claim that rheological stabilization was not the only mechanism involved.

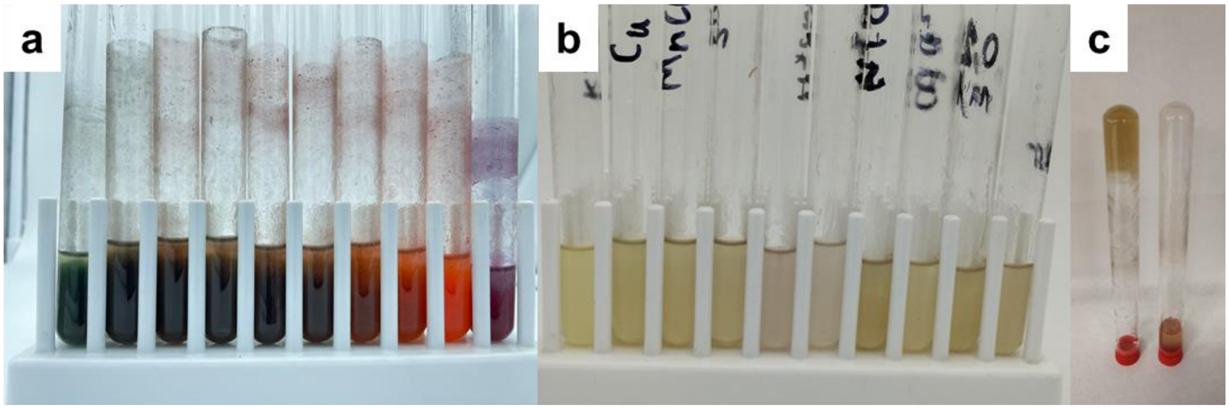

Figure S1. Pictures of nanocellulose-stabilized emulsions of dithizone/chloroform, highlighting extreme cases (a), a post-hoc hypothesis to develop selectivity towards Hg^{2+} (b), and the effect of metal salts on the rheology of emulsions (c).

S2. FTIR spectroscopy and thermogravimetric analysis

Due to how diluted DTZ was in the system, only qualitative information can be obtained from the ATR-FTIR spectra of Figure S2. *Pulp*: Measurements were performed directly on papers made from the same pulp used for CNFs. *CNFs and DTZ+CNFs*: The samples were films (or nanopapers) made by solvent evaporation at 50 °C. In any case, the band at approximately 1570 cm^{-1} can be assigned to C=O stretching, and this is why CNFs obtained by regioselective oxidation (towards carboxylate groups) show strong absorption, whereas the original pulp does not. This band may be overlapped with that associated to the vibration of the azo group of DTZ. Regarding other differences between *CNFs* and *DTZ+CNFs*, certain absorption bands above 1200 cm^{-1} could be due to C–N stretching, and that at 730 cm^{-1}, to N–H bending or to the phenyl rings. The prominent peak at roughly 1000 cm^{-1} is typical of cellulose.

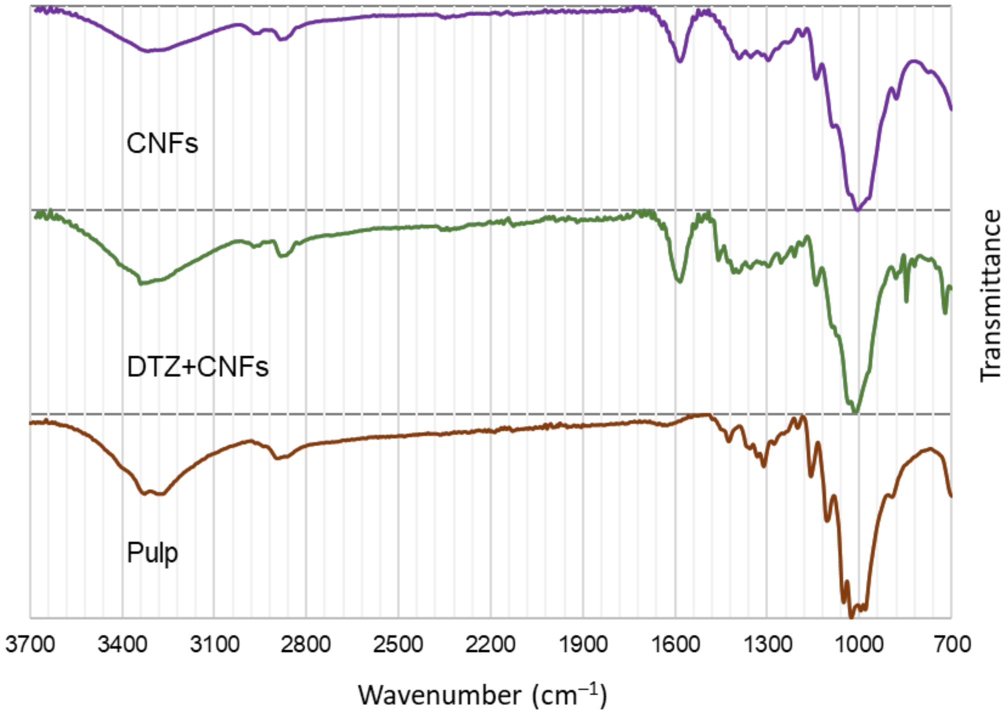

Figure S2. ATR-FTIR spectra. The transmittance has been normalized to the range of display.

The thermal degradation curves in Figure S3 show significant differences. First, the higher mass of ashes at the end of the assay in the case of CNFs can be due to their Na^+ counter-ions, whereas the percentage of organic matter is higher in DTZ+CNFs. The latter sample is also less thermally stable, as its degradation starts at roughly 185 °C. That of CNFs begins at 208 °C. Likewise, the T_{max} of CNFs (the minimum value of the first derivative) is 305 °C. This parameter is as low as 273 °C for DTZ+CNFs.

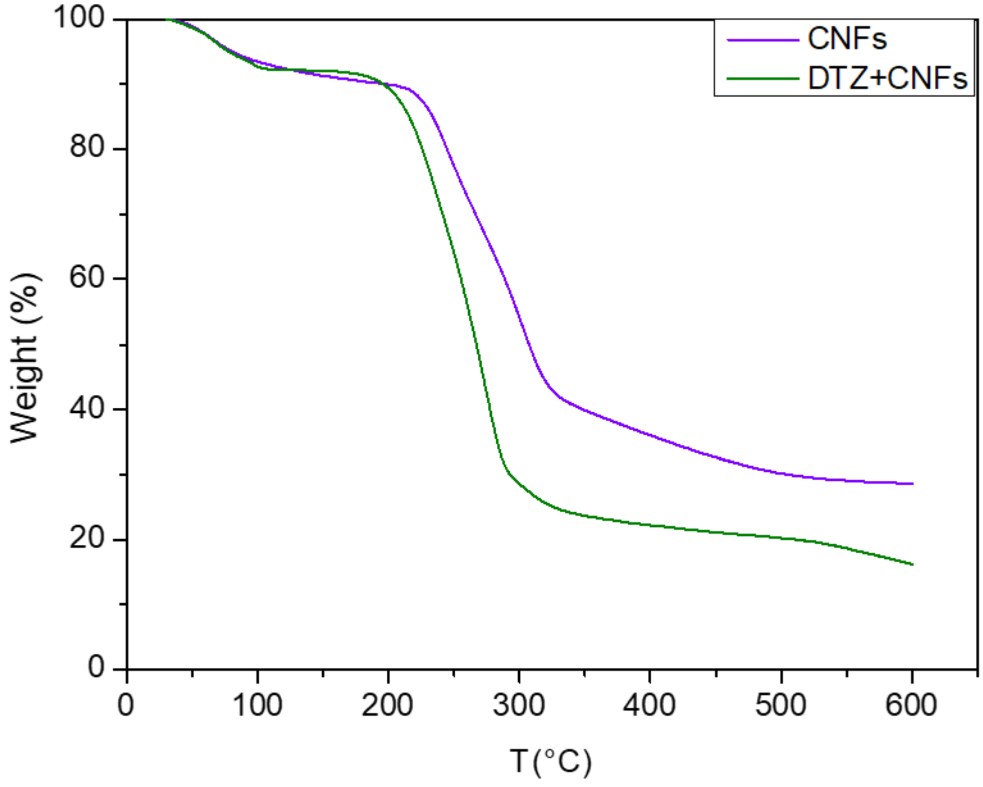

Figure S3. Thermograms of nanocellulose and samples thereof with dithizone.
